# Beyond Targeted Delivery: The Emerging Immunomodulatory Landscape of Antibody-Oligonucleotide Conjugates

**DOI:** 10.3390/ijms27209274

**Published:** 2026-10-19

**Authors:** Yangyang Wang, Shaonan Wang, Huiyan Wang

**Affiliations:** Department of Laboratory Medicine, Jilin Collaborative Innovation Center for Antibody Engineering, Jilin Medical University, Jilin 132013, China

**Keywords:** antibody-oligonucleotide conjugate, adjuvant, innate immunity, neutrophil, immunometabolic reprogramming

## Abstract

Antibody-oligonucleotide conjugates (AOCs) are usually viewed as delivery vehicles that carry nucleic-acid payloads to defined cell populations for gene silencing or gene regulation. Existing reviews concentrate on delivery systems, tumour-directed therapy and extrahepatic oligonucleotide delivery, while the intrinsic immunomodulatory activity of AOCs has received far less attention. Conjugation couples antibody-mediated targeting with the innate immune-stimulating activity of the payload, a property that most current AOCs seldom exploit. This review repositions AOCs as active immune regulators. Three emerging immunoregulatory applications are examined in detail: vaccine-adjuvant construction, neutrophil-driven innate immune activation, and immunometabolic reprogramming. Structure–activity relationships linking AOC design to immune potency are summarized, and their immune mechanisms are distinguished from those of free oligonucleotides and unconjugated antibodies. Based on preclinical and in vitro evidence, AOCs act as multifunctional immunomodulators rather than passive cargo carriers. Two sources of immune activity are distinguished: direct immunostimulation by the conjugated payload, and effects secondary to targeted delivery. These findings still await clinical confirmation. Key barriers to translation, including conjugation efficiency, mechanistic gaps and manufacturing bottlenecks, are discussed. Prospective applications include engineered vaccines, anti-infective innate therapy, tumour microenvironment remodelling and autoimmune intervention, pending further validation. This review provides a conceptual framework for developing next-generation AOC-based immune adjuvants and precision immunotherapeutics.

## 1. Introduction

### 1.1. Opportunities and Clinical Bottlenecks of Oligonucleotide-Based Immunotherapeutics and Monoclonal-Antibody Therapies

Immunostimulatory oligonucleotides such as CpG oligodeoxynucleotide (CpG-ODN) and Stimulator of Interferon Genes agonist (STING-agonist) nucleic acids represent promising candidates for innate immune activation [1]. They initiate robust innate immune cascades by engaging Toll-like receptors (TLR), Retinoic acid-inducible gene I (RIG-I-like) receptors or STING-dependent signalling pathways, and subsequently amplify adaptive immune responses, rendering them attractive vaccine-adjuvant candidates [2,3]. Nevertheless, systemically administered free oligonucleotides are rapidly degraded by nucleases, have short circulation half-lives, and cause non-selective immune-cell activation, raising severe risks such as cytokine-release syndrome and greatly restricting their systemic clinical utility [4].

Monoclonal-antibody immunotherapy has become a cornerstone for tumour and infectious-disease treatment [5]. Leveraging antigen-specific recognition, antibodies precisely target pathological cells [6]; a subset can trigger effector immune responses through Fragment crystallizable gamma (Fcγ) receptor interactions. However, most therapeutic monoclonal antibodies exhibit limited intrinsic innate immune-stimulating capacity and rarely trigger potent, long-lasting innate-to-adaptive immune cascades independently [7].

Adjuvants are the third component of the current immunotherapeutic landscape. Conventional aluminum-salt adjuvants and broad-spectrum nucleic-acid adjuvants can effectively boost immune responses, yet they lack cell-targeting capacity. Conventional aluminum-salt adjuvants and broad-spectrum nucleic-acid adjuvants can effectively boost immune responses, yet they lack cell-targeting capacity. Non-specific immune stimulation frequently leads to local inflammatory reactions and systemic adverse effects [8]. Collectively, oligonucleotides, monoclonal antibodies and traditional adjuvants each possess distinct strengths but suffer from inherent, hard-to-overcome limitations [9,10]. There exists an urgent unmet need to develop novel immunotherapeutics that integrate targeted enrichment and cell-specific immune activation (Table 1). AOCs represent such a class of agents. They combine antibody-mediated cell targeting with payload-driven immune activation.

### 1.2. Rationale Behind AOC Technology: Dual Empowerment of Targeted Delivery and Active Immune Stimulation

**Figure 1:**
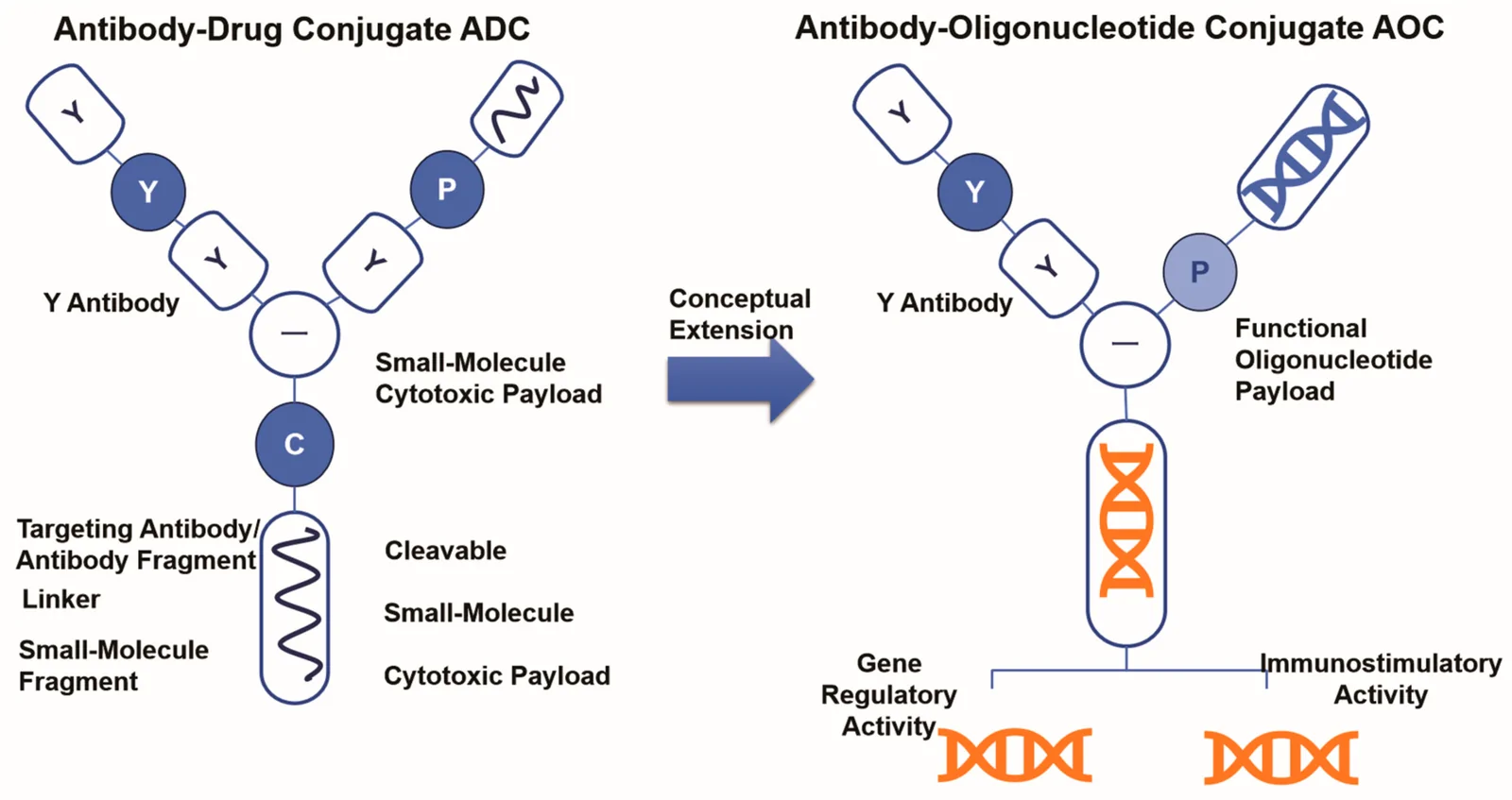
**Structural schematic of ADC and AOC.** Conceptual comparison of ADC and AOC; AOC employs functional oligonucleotide payloads instead of small-molecule cytotoxins.

AOCs are chimeric biomacromolecules chemically assembled from three core modules: a targeting antibody or antibody fragment, a cleavable or non-cleavable linker, and a functional oligonucleotide payload [11]. The antibody performs two functions. It directs the conjugate to target cells. It may also engage Fcγ receptors and contribute to innate immune signals. Conceptually extended from antibody-drug conjugates (ADCs), AOCs replace small-molecule cytotoxic payloads with oligonucleotides capable of gene modulation or immune stimulation (Figure 1).

Historically, AOC development has centred almost exclusively on the “delivery-vehicle” concept: antibodies escort oligonucleotides to target cells, where released nucleic acids exert gene-silencing functions [12]. However, accumulating preclinical evidence indicates that AOCs are far more than passive cargo transporters [11]. Once conjugated to an antibody, immunostimulatory oligonucleotides can be enriched at desired immune-cell populations [13]. Following endocytosis, oligonucleotides are liberated from the conjugate within endolysosomal compartments and activate pattern-recognition receptors, triggering localized immune responses at target sites [14]. Consequently, AOCs combine antibody-mediated targeted enrichment and payload-derived innate immune-stimulating activity, offering a potential strategy to overcome multiple limitations of conventional immunological agents, including poor targeting, high off-target toxicity and insufficient immune-response magnitude [15]. Two contributions should be distinguished. The payload itself drives direct immunostimulation. Targeted delivery raises the local payload concentration and may amplify this activity. The available data do not always separate these contributions; we interpret them separately where the evidence allows.

### 1.3. Current Review Landscape and Existing Research Gaps

Globally, nearly all published AOC-focused studies are confined to optimizing nucleic-acid-delivery efficiency, summarizing siRNA- or antisense-oligonucleotide-mediated gene-silencing therapy and discussing tumour-targeting strategies [16]. Comprehensive studies specifically addressing the intrinsic adjuvant activity of AOCs remain scarce [12]. The immunomodulatory roles of neutrophils in AOC-driven immune responses have rarely been systematically summarized [12,16]. Furthermore, the cross-disciplinary field of AOC-mediated immunometabolic reprogramming is still in its infancy, lacking integrated overviews [12,16]. These knowledge gaps have caused the research community to substantially underestimate AOC potential as active immune regulators and impeded their translational development as vaccine adjuvants and innate immune activators.

### 1.4. Original Perspective of the Present Review

Built upon previous work on vaccine-adjuvant engineering, neutrophil-dependent innate immunity and immunometabolic regulation, the present review breaks away from the traditional delivery-centred framework and establishes a novel conceptual landscape for AOC immunomodulation [17,18,19]. Unlike previous reviews that centred on AOC delivery efficiency, gene-silencing functions and tumour-targeting strategies, this review focuses on the intrinsic immune activity of AOCs. We dissect multi-faceted immune functions of AOCs across three pillars: vaccine-adjuvant effects, neutrophil-mediated innate immune cascades and immunometabolic reprogramming. Disease-specific application prospects, mechanistic uncertainties and translational obstacles are discussed, providing guidance for the future development of immune-targeted AOC therapeutics.

## 2. Structural Classification and Fundamental Mechanisms of AOCs

This chapter provides concise foundational background while deliberately avoiding redundant content already extensively covered in delivery-oriented reviews, with special emphasis on structure-immunogenicity relationships.

### 2.1. Dominant Structural-Design Strategies for AOCs

Three indispensable building blocks constitute each AOC construct: the antibody module, the linker and the oligonucleotide payload.

(1) Antibody module: Full-length Immunoglobulin G (IgG), Fab fragments or single-chain variable fragments (scFv) are commonly adopted [11]. Full-length IgG molecules retain Fc-region domains capable of engaging Fcγ receptors on immune cells, which may trigger additional innate immune signals [11,16]. By contrast, Fab and scFv fragments lack Fc domains, thereby eliminating Fc-dependent non-antigen-specific immune-cell activation and lowering off-target inflammatory risks [20]. Target antigens can be selected from dendritic-cell surface markers, neutrophil-specific receptors or tumour-associated antigens to define the cellular-targeting preference of each AOC [11]. Fcγ receptor engagement has two faces. On target cells, it can amplify desired immune activation [21]. On bystander cells, it can cause non-antigen-specific stimulation [22]. The net effect depends on the antibody isotype, the target antigen and the dose [23].

(2) Linker: Linkers are categorized as cleavable or non-cleavable. Cleavable linkers undergo hydrolysis in acidic endosomal environments, lysosomal protease-rich compartments or intracellular reductive conditions, liberating free oligonucleotide payloads and are therefore widely used for TLR-activation applications [24]. Non-cleavable linkers confer superior conjugate stability and are generally preferred for gene-silencing AOCs [11,16]. Linker length, hydrophobicity and cleavage efficiency critically determine whether oligonucleotides can be successfully released to activate endosomal immune receptors [14].

(3) Oligonucleotide payload: Beyond siRNA and antisense oligonucleotides designed for gene knockdown, immunostimulatory sequences such as CpG-ODN and STING-agonist nucleic acids serve as core payloads that endow AOCs with adjuvant properties [9].

Conjugation chemistry falls into two broad categories: random conjugation and site-specific conjugation [25]. Conjugation sites include solvent-exposed lysine residues and engineered cysteine residues [26]. Site-specific methods use enzymatic ligation or non-natural amino acids. The drug-to-antibody ratio (DAR) usually ranges from 1 to 4 for AOCs [27]. Conjugation site and DAR determine payload stoichiometry, conjugate stability and immune potency [28]. Site-specific conjugation gives uniform conjugates [29]. Uniformity improves batch-to-batch reproducibility and simplifies quality control. Random conjugation produces heterogeneous species [30]. Heterogeneity can reduce stability and increase off-target effects [31]. For immunomodulatory AOCs, site-specific methods are therefore preferred [30,31,32].

### 2.2. Structure-Immunological-Activity Relationships

The magnitude of AOC-triggered immune activation is highly dependent on overall molecular architecture [14]. Antibody target selection dictates which immune-cell subsets receive concentrated immunostimulatory signals [33]. Excessively high DARs frequently cause molecular aggregation and accelerated in vivo clearance, whereas insufficient payload density results in weak immune stimulation [16]. Inefficient linker cleavage prevents oligonucleotide dissociation from the conjugate backbone, blocking access to endosomal TLRs and abrogating adjuvant activity [34].

Accordingly, immunomodulatory AOCs cannot directly adopt design parameters optimized for conventional gene-delivery constructs [12,35]. Structure-optimization workflows for immune-regulating AOCs should use immunological readouts as primary evaluation endpoints [34,36]. Antibody conjugation changes how immune cells recognize the oligonucleotide payload. The antibody directs uptake through receptor-mediated endocytosis [14]. This increases delivery to endosomal compartments, where TLR3, TLR7/8 and TLR9 reside [14,37]. Conjugation limits cytosolic access [14]. Free oligonucleotides can reach cytosolic sensors such as RIG-I and STING [38]; conjugated payloads remain in endolysosomes until linker cleavage [39]. The antibody itself can engage Fcγ receptors and add innate signals [40]. The net cytokine output thus depends on the antibody, the linker and the payload [41,42,43].

### 2.3. Brief Overview of Canonical Targeted-Delivery Functions of AOCs

**Figure 2:**
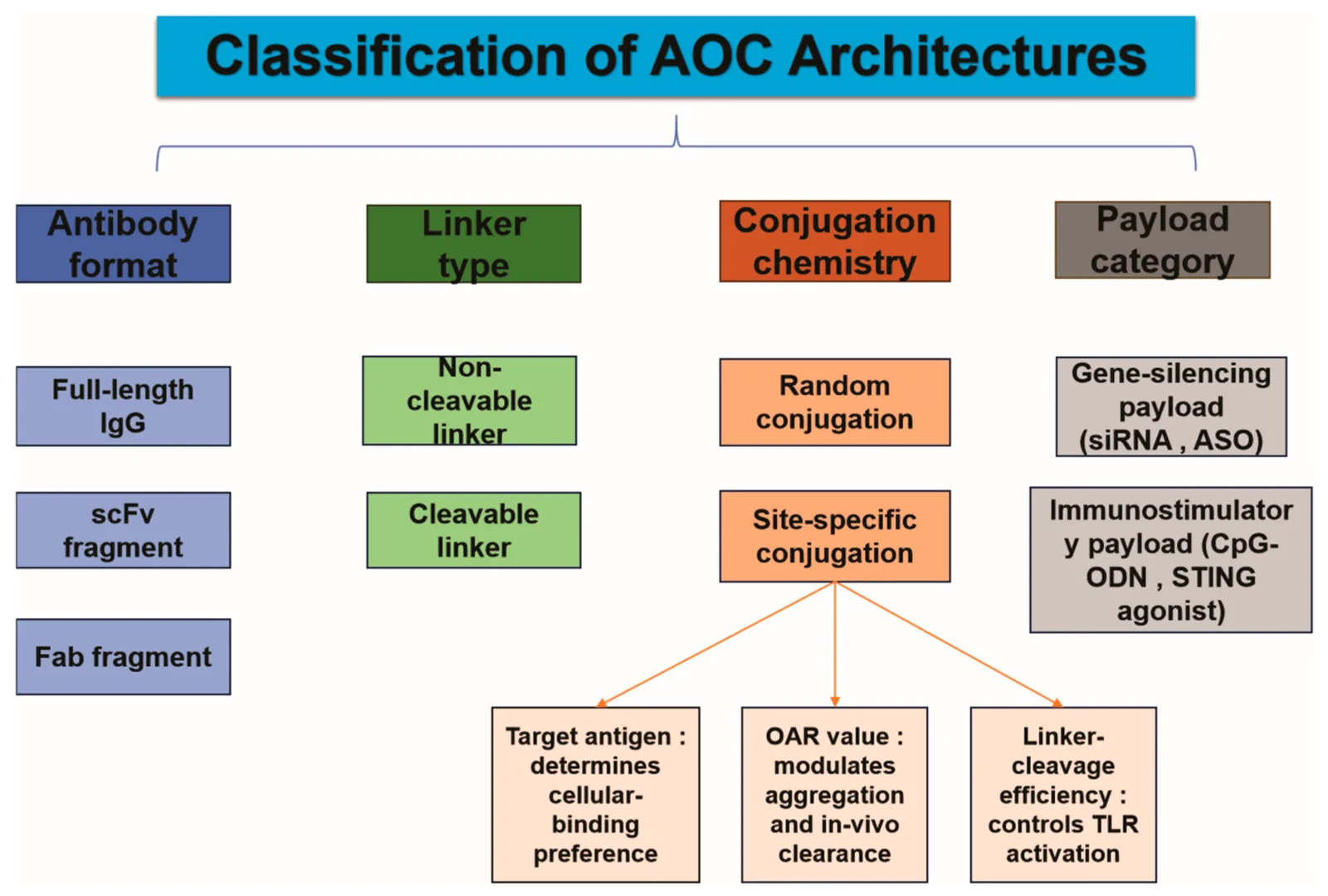
**Classification scheme of AOC architectures.** Antibody format, linker type, conjugation site, payload class of AOC molecules. scFv, single-chain variable fragment.

The most well-established function of traditional AOCs lies in targeted nucleic-acid delivery. Following antigen-mediated binding and endocytic internalization [12,15], oligonucleotide payloads are released intracellularly to silence target-gene expression [12,15,35]. Since multiple excellent studies have exhaustively covered this topic, it will not be elaborated herein [15,35]. Subsequent chapters focus predominantly on under-explored immunomodulatory properties (Figure 2).

## 3. Vaccine-Adjuvant Effects and Underlying Molecular Mechanisms of AOCs

### 3.1. Intrinsic Adjuvant Potential of Oligonucleotide Payloads

**Figure 3:**
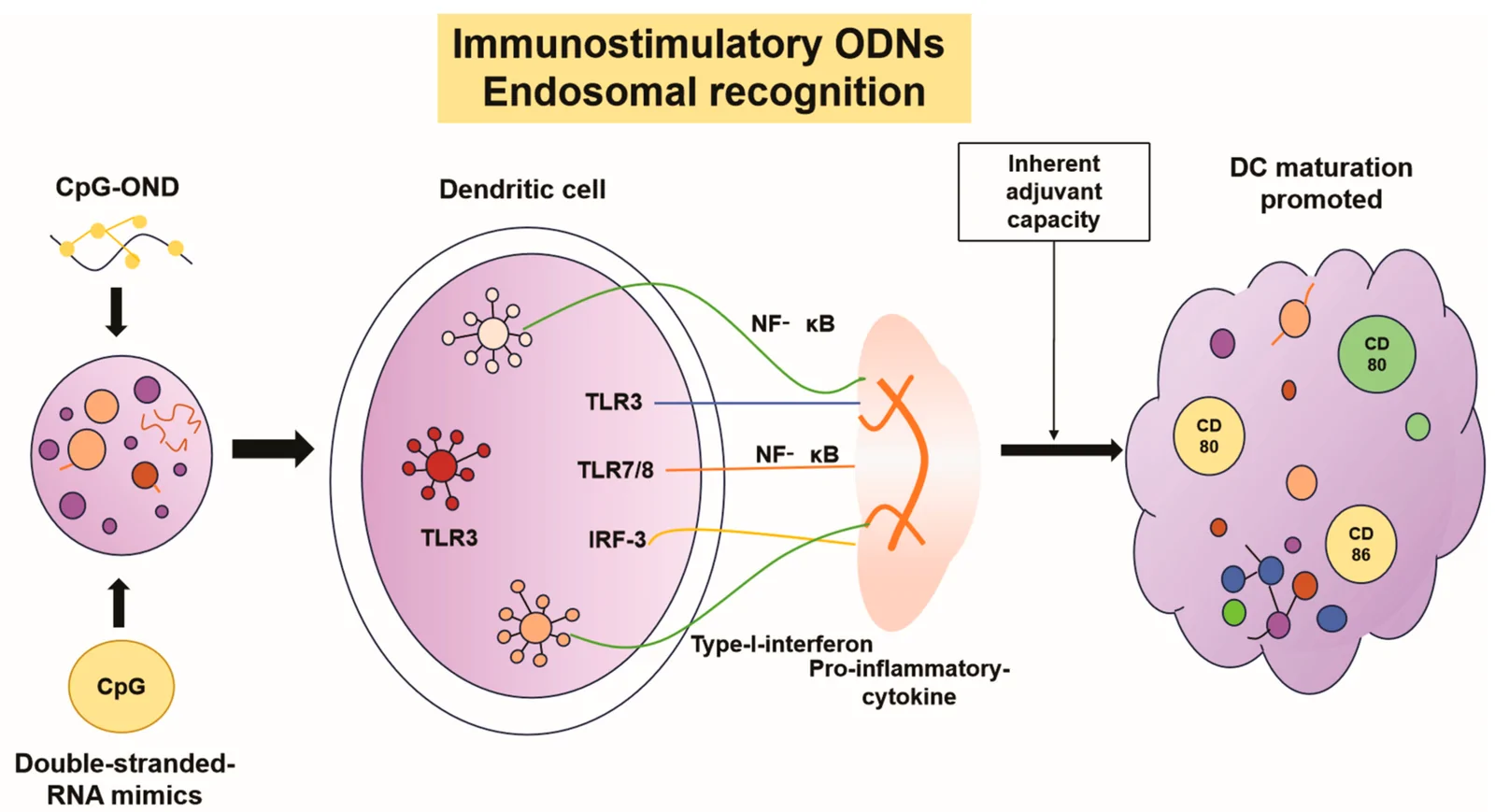
**Innate immune activation by endosomal-sensing immunostimulatory oligonucleotides.** CpG-ODN and dsRNA mimics are recognized by endosomal TLR9, TLR3, and TLR7/8, initiating NF-kappaB and IRF-3 cascades. Arrows indicate signalling direction. DC, dendritic cell; TLR, Toll-like receptor; NF-kappaB, nuclear factor kappa-light-chain-enhancer of activated B cells; IRF-3, Interferon Regulatory Factor 3; CD80/CD86, co-stimulatory molecules.

Once internalized into endosomes of immune cells, immunostimulatory oligonucleotides such as CpG-ODN and double-stranded RNA mimics are recognized by endosomal Toll-like receptor 9 (TLR9), Toll-like receptor 3 (TLR3), Toll-like receptor 7/8 (TLR7/8) and other pattern-recognition receptors [3,44,45]. Downstream Nuclear factor-κB (NF-κB) and interferon regulatory factor 3 (IRF-3) signalling cascades are initiated, inducing type-I interferon and pro-inflammatory cytokine secretion [46,47]. Dendritic-cell maturation is promoted, accompanied by upregulation of Cluster of Differentiation 80 (CD80) and Cluster of Differentiation 86 (CD86) co-stimulatory molecules, demonstrating the inherent adjuvant capacity of immunostimulatory nucleic acids [48]. However, free oligonucleotides lack cell-targeting specificity and exhibit a narrow therapeutic safety window upon systemic administration (Figure 3).

### 3.2. Antibody-Mediated Targeted Enrichment Enables Cell-Specific Adjuvant Activation

The antibody moiety grants AOCs precise targeting capacity, enabling selective delivery of immunostimulatory oligonucleotides to antigen-presenting-cell populations such as dendritic cells [13]. For instance, CLEC9A-targeted AOC-CpG conjugates concentrate CpG payloads within cross-presenting conventional type-1 dendritic cell subsets, triggering localized immune stimulation while avoiding widespread systemic immune activation and associated adverse events [49]. This “targeted delivery plus in situ immune triggering” paradigm constitutes the core advantage of AOCs relative to uncoupled free-oligonucleotide adjuvants.

### 3.3. Synergistic Adjuvant Mechanisms of AOCs in Genetically Engineered Vaccines

When co-administered with recombinant-protein or subunit vaccines, AOCs exert powerful synergistic adjuvant effects (Table 2) [50,51]. After targeted enrichment and activation of antigen-presenting cells, mature dendritic cells efficiently capture, process and present vaccine antigens via Major Histocompatibility Complex class I (MHC-I) and Major Histocompatibility Complex class II (MHC-II) molecules [52,53]. Concurrent type-I-interferon secretion enhances antigen cross-presentation and drives robust CD8^+^ cytotoxic-T-cell and CD4^+^ helper-T-cell responses [54]. Compared with free nucleic-acid adjuvants, targeted AOC adjuvants can achieve stronger vaccine-induced immunity at substantially reduced dosages [55].

### 3.4. Preclinical Comparison Between AOC Adjuvants and Traditional Adjuvants

Table 3 summarizes the comparison of AOCs, free oligonucleotides and unconjugated antibodies. Direct head-to-head data are scarce. The comparison reflects the evidence discussed in this review and should be read with that limitation in mind.

## 4. Neutrophil-Mediated Innate Immune Regulation by AOCs

### 4.1. Research Gap: Neutrophils Have Long Been Overlooked in AOC-Immunology Studies

Previous immunological investigations of AOCs have overwhelmingly focused on dendritic cells and macrophages as primary effector populations [59]. Neutrophils, the most abundant innate immune-cell subset among circulating leukocytes in humans, have been largely neglected within the AOC research field [60]. Beyond their well-known front-line anti-pathogen functions, neutrophils release neutrophil extracellular traps (NETs) and secrete extensive cytokine panels, orchestrating subsequent dendritic-cell and T-cell responses while contributing to immune-memory formation [61]. Neutrophils therefore represent a functionally important effector population mediating AOC-driven immune regulation.

Neutrophil activation is not always beneficial. Excessive NET formation can promote thrombosis, tissue damage and sterile inflammation [62]. Sustained neutrophil recruitment may maintain chronic inflammatory microenvironments [63]. These adverse effects must be considered when designing neutrophil-targeted AOCs. They also underline the need for controlled, transient neutrophil activation.

Based on mechanistic reasoning, neutrophil-receptor-targeted AOCs are predicted to drive neutrophil activation, recruitment and tissue infiltration; however, no published study has yet confirmed this using a fully assembled AOC construct. Neutrophil-receptor-targeted AOC constructs can be recognized and internalized by neutrophils. Intracellularly released oligonucleotide payloads activate TLR-dependent signalling pathways and trigger neutrophil activation [64]. Activated neutrophils upregulate chemokine-receptor expression, promoting neutrophil recruitment and tissue infiltration towards lymph nodes, infection foci or tumour sites [65,66]. In turn, stimulated neutrophils secrete chemoattractants that recruit additional immune-cell populations and amplify local innate immune inflammatory microenvironments [67,68].

Neutrophil-dependent regulation of inflammatory responses and immune memory is well established for free TLR agonists [69], and we propose that AOC-mediated targeting could extend these effects to localized settings. Direct evidence with AOC constructs is not yet available. AOC-activated neutrophils secrete pro-inflammatory cytokines including Tumour Necrosis Factor-α (TNF-α) and Interleukin-1β (IL-1β), shaping local inflammatory cascades [70]. Cell-to-cell crosstalk between stimulated neutrophils and dendritic cells accelerates dendritic-cell maturation and antigen presentation, indirectly potentiating adaptive immune responses [71]. Emerging evidence indicates that specialized neutrophil subpopulations participate in the establishment of long-term immune memory, suggesting that neutrophil-targeted AOCs could extend vaccine-mediated immune protection [72].

### 4.2. Translational Potential of Neutrophil–AOC Immune Axes in Anti-Infective and Tumour Immunology

**Figure 4:**
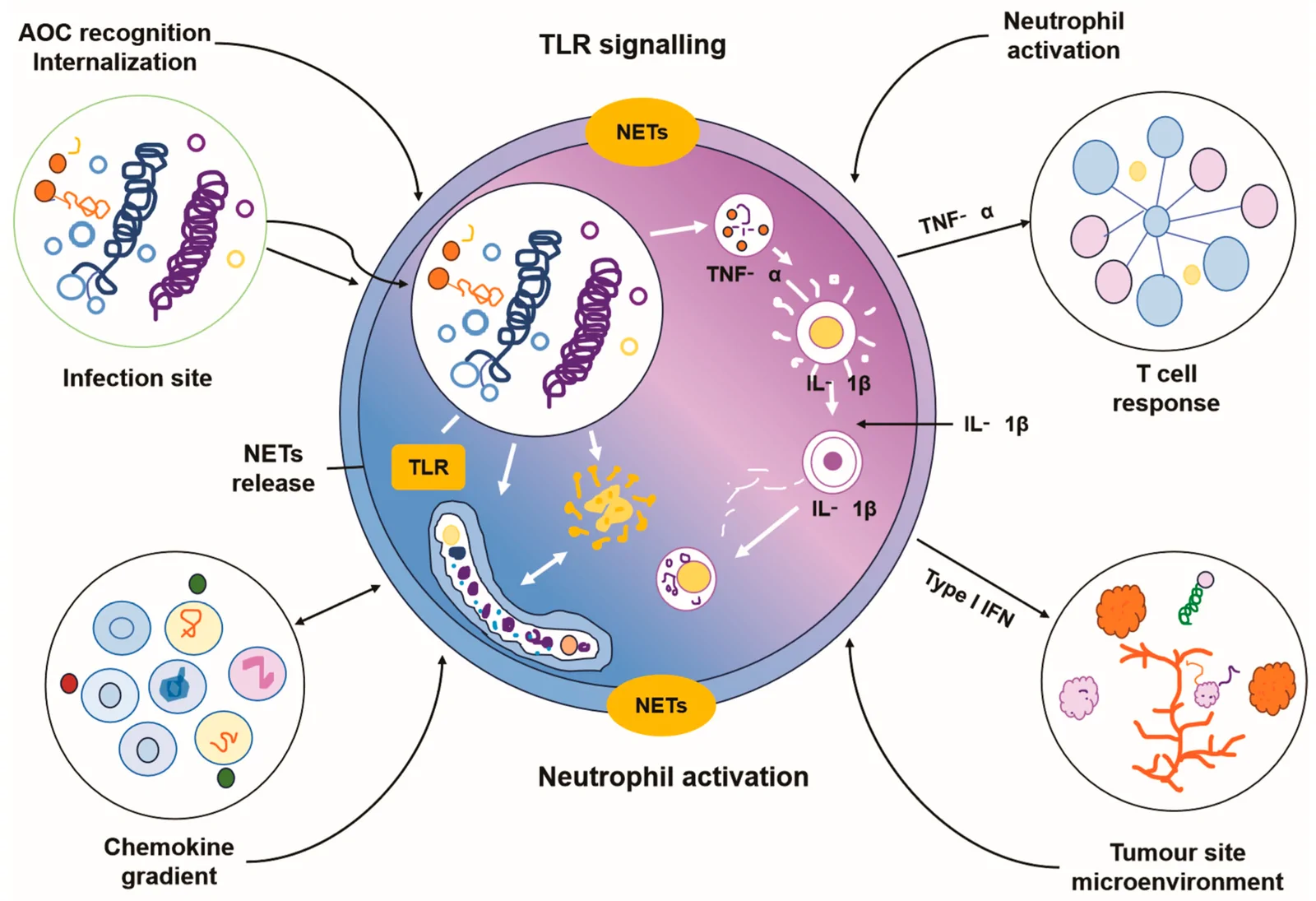
**Proposed model of neutrophil-centred innate immune signalling following AOC engagement.** Hypothetical pathway: AOC-delivered TLR signalling in neutrophils may induce NET formation and pro-inflammatory mediators, potentially promoting DC maturation and downstream adaptive immunity within infectious or tumour microenvironments. Abbreviations: NET, neutrophil extracellular trap; TLR, Toll-like receptor.

In bacterial and viral infection contexts, neutrophil-targeted AOCs are hypothesized to selectively activate neutrophils at disease sites, enhancing early-stage host innate immune defence. The cited evidence [73] describes innate immune priming by a detergent-based adjuvant, not by an AOC construct. Translation to AOC-specific neutrophil activation awaits experimental validation. Within tumour-immunology applications, AOC-based immunomodulation is envisioned to reprogramme tumour-associated neutrophils from pro-tumour N2 towards anti-tumour N1 phenotypes [74]. This concept is extrapolated from Smad3-dependent TAN polarization studies [74] and has not been tested with any AOC construct. Combined administration with cancer vaccines or immune-checkpoint inhibitors may yield synergistic anti-tumour effects [75]. Collectively, neutrophil-targeted AOCs represent an innovative therapeutic strategy for infectious diseases and cancer (Figure 4).

## 5. Core Mechanisms Underlying AOC-Triggered Immunometabolic Reprogramming

### 5.1. Intrinsic Linkage Between Immunometabolic Reprogramming and Immune-Cell Activation

Immune-cell functional states are tightly coupled to intracellular metabolic patterns. Quiescent immune cells rely primarily on oxidative phosphorylation for energy supply [76]. Following TLR-mediated activation, dendritic cells, neutrophils and other immune-cell subsets rapidly upregulate aerobic glycolysis while remodelling lipid and glutamine metabolism [77]. Metabolic reprogramming provides essential biomaterials to support immune-cell maturation, cytokine production and effector function [78,79]. Conversely, metabolic changes modulate transcription-factor activity, determining immune-response magnitude, duration and polarization profiles [80,81]. Metabolic-pathway manipulation has therefore become a cutting-edge strategy for next-generation immunotherapeutic design [82]. Two caveats apply. Most immunometabolic studies used free TLR agonists or activated immune cells. Direct evidence that AOC treatment reprograms immune-cell metabolism is still scarce. The model presented below is therefore a working hypothesis.

AOC-mediated modulation of glycolysis, lipid metabolism and glutaminolysis in immune cells has been proposed as below. Direct evidence in AOC-treated cells is still limited; the following model is based largely on studies of free immunostimulatory oligonucleotides and TLR-activated immune cells (Level 2: inferred evidence). Following endocytic uptake by target immune cells, oligonucleotide payloads from internalized AOCs are hypothesized to activate TLR-NF-κB signalling cascades, upregulating metabolic transcription factors such as HIF-1α [83]. This transcriptional switch is proposed to drive a metabolic transition from oxidative phosphorylation to aerobic glycolysis, enhancing cellular glucose uptake and lactate production. Direct demonstration of these metabolic changes following AOC treatment (Level 1: experimental evidence) remains to be established [84]. Meanwhile, analogous immune-stimulatory signals have been shown to re-balance intracellular fatty acid synthesis and oxidation, and to increase glutamine-transporter expression in macrophages and neutrophils [85,86,87]. Whether AOC-delivered payloads produce these same metabolic effects in target cells has not been demonstrated. Coordinated remodelling across these metabolic pathways collectively establishes the AOC-induced immunometabolic phenotype (Table 4).

### 5.2. Immunometabolic Reprogramming Explains Prolonged Adjuvant Effects of AOCs

Free oligonucleotide adjuvants frequently induce only transient immune-cell activation with rapidly decaying immune responses [58]. By contrast, targeted AOCs may deliver sustained localized immunostimulation, promoting more stable metabolic reprogramming in recipient immune cells [88,89,90]. The metabolically reprogrammed cellular phenotype may extend cytokine-secretion windows and generate durable adjuvant effects. This immunometabolic mechanism offers a theoretical explanation for the longer-lasting immune-stimulatory potency of AOCs compared with free oligonucleotides. This explanation remains hypothetical until validated in AOC-treated immune cells.

**Figure 5:**
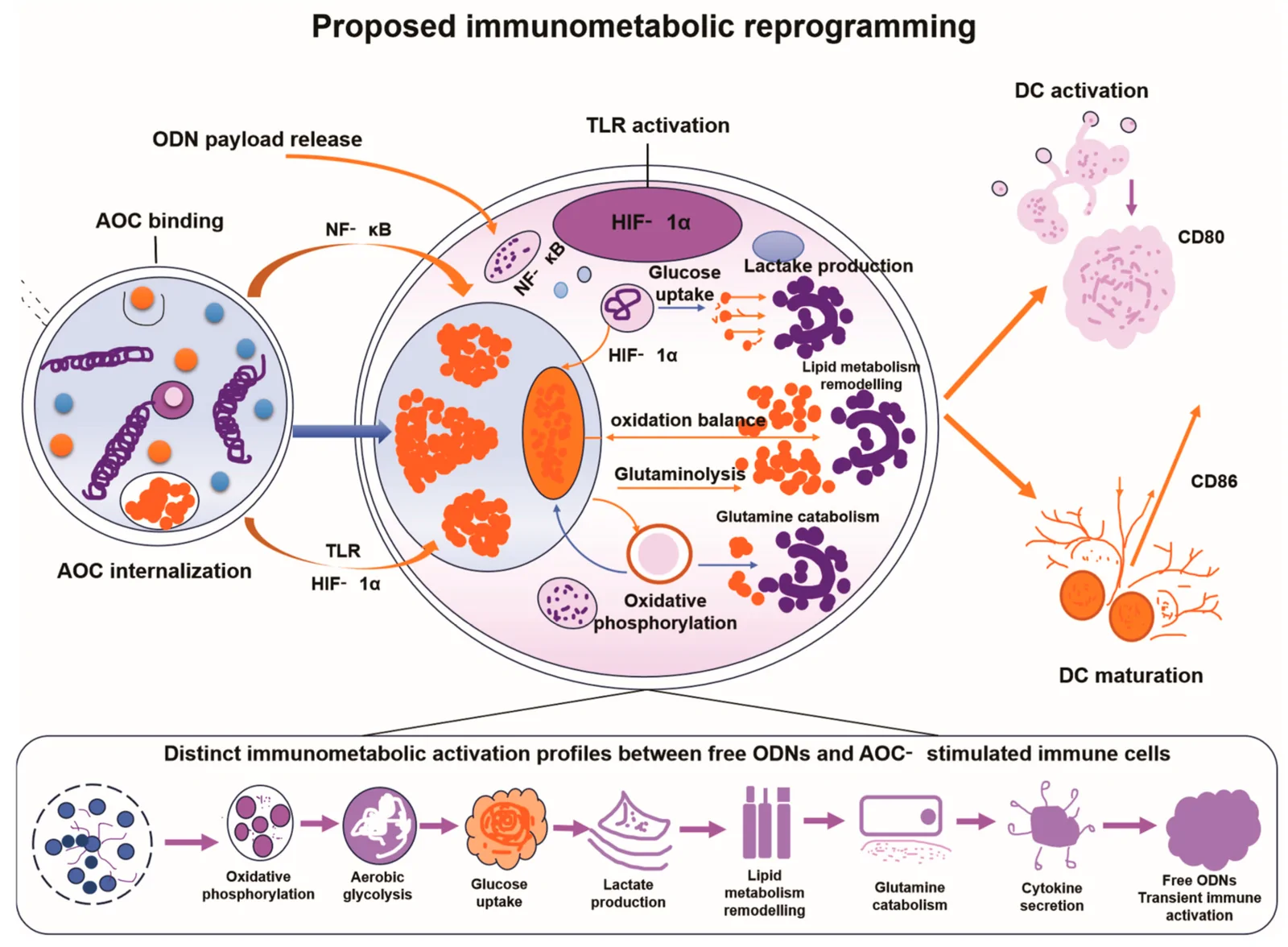
**Proposed immunometabolic reprogramming following AOC stimulation.** The TLR-NF-kappaB-HIF-1alpha axis remodels glycolysis, lipid, and glutamine metabolism, facilitating DC maturation. DC, dendritic cell; HIF-1alpha, hypoxia-inducible factor 1 alpha; NF-kappaB, nuclear factor kappa-B; TLR, Toll-like receptor.

Rational optimization strategies for AOC immunopotency are guided by immunometabolism. In future preclinical workflows, immunometabolic biomarkers may be incorporated as quantitative readouts for evaluating AOC immunological activity [91]. Rational structural modifications can be introduced to engineer AOCs that selectively drive Th1-favoured immunometabolic profiles [92,93]. Dual-functional AOC constructs, combining pattern-recognition-receptor agonism and immunometabolic-pathway modulation, may be generated by conjugating metabolism-regulating oligonucleotides to targeting antibodies, enabling finely tuned, predictable immune outputs (Figure 5).

## 6. Multifaceted Application Prospects of AOC-Mediated Immunomodulation

### Development of Targeted Adjuvants for Genetically Engineered Vaccines

Targeted vaccine-adjuvant development represents one of the most translation-ready applications of the AOC platform. Dendritic-cell-targeted AOC adjuvants, co-formulated with recombinant-protein, peptide or mRNA vaccines, can induce potent and durable Th1-biassed cellular immunity at reduced doses while minimizing systemic side effects, offering next-generation adjuvant solutions for anti-infective and therapeutic cancer vaccines [55].

AOC-based innate immune activation therapy may treat infectious diseases. Neutrophil- or macrophage-targeted AOC therapeutics can trigger localized innate immune activation at infection sites, accelerating early pathogen clearance during acute bacterial or viral illnesses [94]. Such immune-boosting agents may serve as valuable immunotherapeutic interventions against infectious diseases.

**Figure 6:**
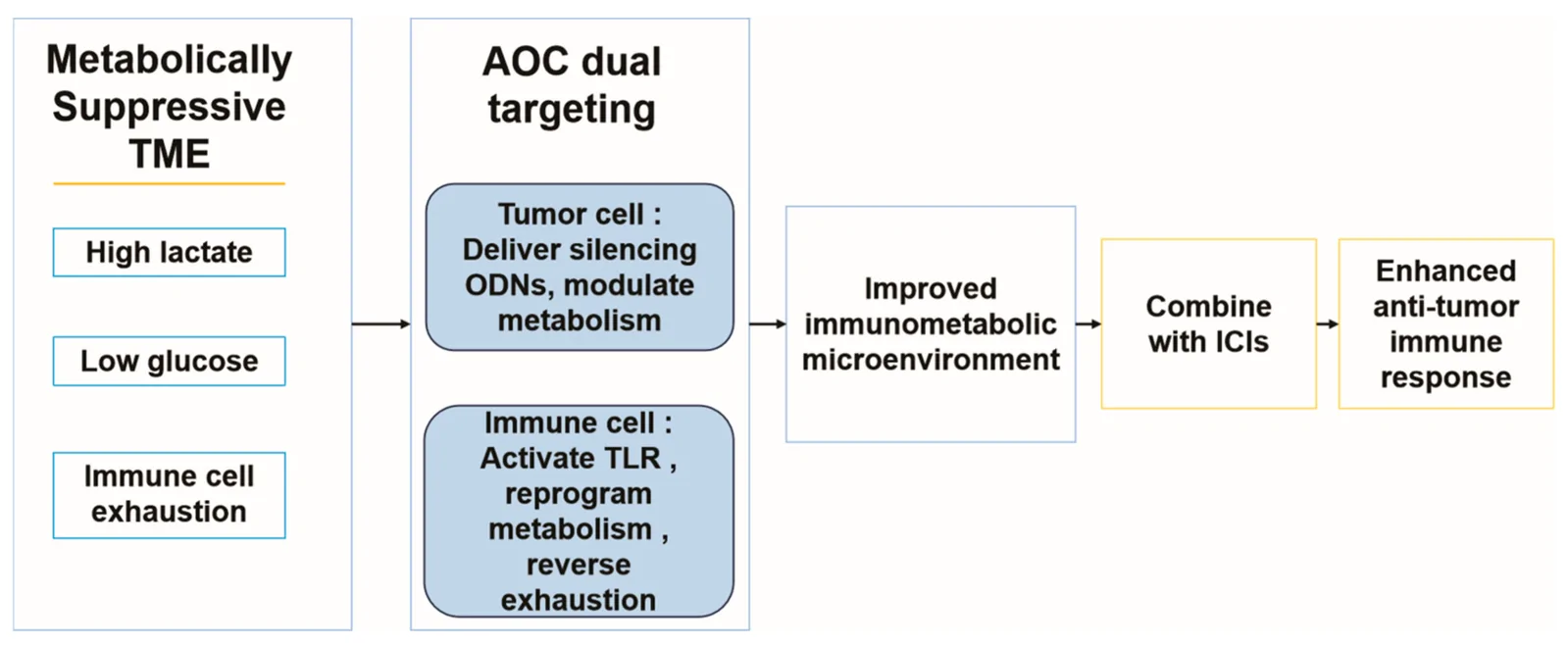
**AOC-driven tumour microenvironment (TME) remodelling for immune-checkpoint inhibitor (ICI) combination therapy.** Dual-targeting AOCs reverse immune-cell exhaustion within the metabolically suppressive TME (glucose-deprived, lactate-rich) and potentiate ICI-mediated anti-tumour immunity. TME, tumour microenvironment; ICI, immune-checkpoint inhibitor.

Metabolic modulation and immune remodelling of the tumour microenvironment. The tumour microenvironment is characterized by metabolically suppressive conditions, including glucose deprivation and excessive lactate accumulation [95]. Beyond direct tumour-cell-targeted gene silencing, AOCs can be engineered to target tumour-infiltrating immune cells and reverse immune-cell exhaustion via immunometabolic reprogramming, restoring anti-tumour immune function and generating synergistic therapeutic benefits when combined with immune-checkpoint inhibitors (Figure 6).

AOCs may also intervene in autoimmune diseases. Notably, certain oligonucleotide payloads exert immunosuppressive effects. By altering oligonucleotide sequences and selecting distinct cellular targets, AOCs can suppress over-activated immune cascades and re-establish immune tolerance in autoimmune-disease contexts [11,96]. Candidate target populations include dendritic cells, B cells and regulatory T cells. AOCs directed to tolerogenic dendritic cells may promote regulatory T-cell induction. Targeting B cells may reduce autoantibody production. These strategies could restore immune tolerance with less systemic immunosuppression than current therapies. This bidirectional regulatory capacity substantially broadens the application scope of immunomodulatory AOC platforms.

To date, no antibody-oligonucleotide conjugate has received regulatory approval specifically for an immunomodulatory indication. Several AOC programmes have entered early-phase clinical trials (e.g., AOC 1001, AOC 1020 for CNS indications), but these focus on gene-silencing payloads rather than immune activation. ISAC-specific candidates remain at the preclinical or IND-enabling stage. This translational gap underscores the exploratory nature of the applications discussed below.

## 7. Current Bottlenecks and Challenges Facing Immunomodulatory-AOC Research

### 7.1. Structural-Optimization Challenges: Conjugation Efficiency, In Vivo Stability and Off-Target Immune Risks

Immunomodulatory AOC development faces multiple technical manufacturing obstacles. Site-specific conjugation protocols are labour-intensive and difficult to scale up economically [97,98]. Payload shedding from circulating AOCs may release free oligonucleotides and trigger undesired off-target inflammation [99]. Fcγ receptor engagement by full-length IgG-based AOCs represents an additional source of unintended non-antigen-specific immune stimulation [34]. Free oligonucleotides are strong TLR agonists [44]. Their systemic release can cause non-selective immune activation and cytokine release. Payload release may also reduce the intended local adjuvant effect. Linker stability is therefore a key design parameter for immunomodulatory AOCs.

AOCs also carry platform-specific safety risks. Cytokine-release syndrome remains possible when large amounts of potent immunostimulatory payloads are released [9]. Conjugated nucleic acids may activate complement [100]. Off-target uptake in scavenger cells, such as liver Kupffer cells and spleen macrophages, can concentrate immune signals in immune organs [101]. The antibody component can be immunogenic and induce anti-drug antibodies [9]. Repeated dosing may cause prolonged immune stimulation and chronic inflammation. These risks require dedicated preclinical evaluation.

### 7.2. Mechanistic Limitations: Incomplete Neutrophil-Immunometabolic Regulatory Networks

Existing experimental evidence describing neutrophil–AOC interactions is largely limited to phenotypic observations of neutrophil activation and recruitment [14]. Complete downstream signalling and immunometabolic cascades remain incompletely mapped [102]. Real-time in vivo temporal dynamics of multi-cell immune cascades involving neutrophils, dendritic cells and lymphocytes following AOC administration have rarely been visualized [103,104]. Key upstream and downstream molecular nodes governing AOC-driven immunometabolic reprogramming await further identification [9,14]. These mechanistic uncertainties hinder rational, precision-guided AOC design.

These gaps have technical origins. Current experimental models do not fully reproduce neutrophil biology [105]. Live imaging of neutrophil–AOC interactions in vivo is difficult [106]. Molecular tools to track conjugated payloads at the single-cell level are still limited.

### 7.3. Translational Barriers: Large-Scale Manufacturing, Biosafety and Clinical Compatibility

**Figure 7:**
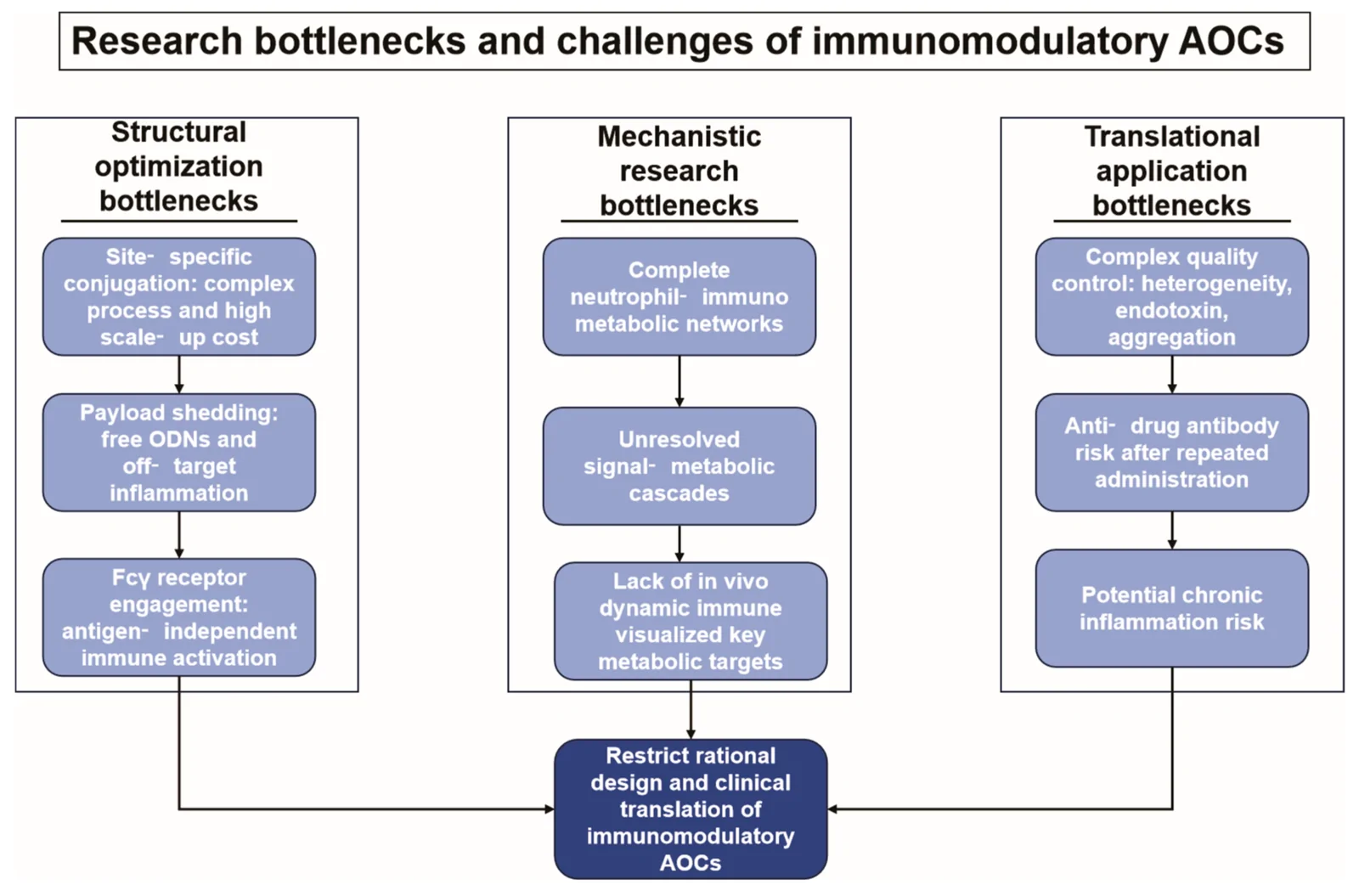
**Key challenges limiting immunomodulatory AOC translation.** Three bottleneck categories: (i) structural—conjugation heterogeneity, payload shedding, Fc-mediated off-target activation; (ii) mechanistic—incomplete neutrophil signalling maps, unvalidated immunometabolic pathways; (iii) translational—manufacturing scalability, ADA risk, absence of clinical-stage immunomodulatory AOCs. Fc, fragment crystallizable.

As complex chimeric biomacromolecules, AOCs require highly specialized quality-control frameworks to monitor product heterogeneity, endotoxin contamination and macromolecular aggregation [25]. Anti-drug-antibody formation following repeated AOC administration and chronic-inflammation risks from sustained immune activation must be comprehensively assessed through dedicated toxicology studies [16]. No AOC has been approved by the FDA to date. Gene-silencing AOCs, such as AOC 1001 and AOC 1044, are in clinical trials (Table 5). The immunomodulatory AOCs discussed in this review remain at the preclinical stage. Multiple translational hurdles remain before immunomodulatory AOCs can advance to human clinical trials (Figure 7). Repeated dosing may also induce antibodies against the antibody component or the linker-payload part. These anti-drug antibodies can reduce efficacy and cause infusion reactions. Long-term immunogenicity data are not yet available for immunomodulatory AOCs.

## 8. Discussion

AOCs deliver immunostimulatory payloads to defined antigen-presenting-cell populations. They elicit balanced Th1-type cellular and humoral responses at lower doses than aluminum or free nucleic-acid adjuvants. Neutrophil-directed AOCs trigger TLR-dependent activation, recruitment and tissue infiltration, and shape downstream dendritic-cell and T-cell responses. Some evidence links neutrophil subsets to immune memory. The immunometabolic dimension connects acute innate stimulation to durable immune function. Internalized payloads activate TLR-NF-κB-HIF-1α signalling and shift recipient cells toward aerobic glycolysis and glutamine metabolism. Taken together, these mechanisms broaden the clinical reach of the platform. Applications range from engineered vaccines and anti-infective innate therapy to tumour microenvironment remodelling and autoimmune intervention. The conclusions remain constrained, however. Most evidence is preclinical. Only a few studies provide direct experimental data on AOC-driven immunomodulation; other claims rest on free-oligonucleotide studies or indirect observations (Table 6). Fc-mediated off-target activation, payload shedding, conjugation scalability and product quality control also need to be resolved. These gaps define the near-term agenda: structure-based design guided by immune and immunometabolic endpoints, dedicated neutrophil-targeted platforms, and multi-target combinations with immune-checkpoint inhibitors. If these are addressed, the immunomodulatory view of AOCs developed here can be tested in settings where current adjuvants and innate immune therapies fall short. Two levels of evidence should be separated. Payload-driven TLR activation and dendritic-cell maturation are supported by direct experiments. The coordinated innate-to-adaptive cascade and the immunometabolic connection are largely inferred.

## 9. Conclusions and Future Perspectives

AOCs have long been treated as passive carriers for targeted nucleic-acid delivery. On the basis of preclinical evidence, this review proposes that they act as multifunctional immunomodulatory biomolecules. Direct clinical validation is still lacking. Through antibody-mediated cellular enrichment and the intrinsic activity of their payloads, AOCs elicit vaccine-adjuvant effects, initiate neutrophil-driven innate immune cascades and induce immunometabolic reprogramming. These three mechanisms act in concert to shape integrated innate-to-adaptive immune responses. Future work should advance along four directions. Structure-based design of AOC vaccine adjuvants should be guided by immunometabolic readouts. Targeting moieties, linkers and payloads should be tuned for durable, controllable adjuvant immunity rather than gene-silencing efficiency alone. Neutrophil-specific platforms depend on a fuller understanding of AOC-driven activation, NET release and phenotypic polarization. Dedicated surface biomarkers should be screened for infectious-disease and oncology applications. Multi-target strategies should co-engage antigen-presenting cells and neutrophils and combine AOC adjuvants with immune-checkpoint inhibitors and metabolic modulators. In parallel, scalable site-specific conjugation, dedicated quality-control specifications and systematic preclinical safety assessment are needed to move immunomodulatory AOCs toward human clinical trials. Despite remaining mechanistic gaps and translational obstacles, research centred on AOC immunomodulation will advance the development of next-generation targeted immune adjuvants and precision immunotherapeutics. The most critical gaps are mechanistic validation, scalable manufacturing and human safety data.

## Figures and Tables

**Table 1:** **Advantages and limitations of representative immunotherapeutic agents.** Comparison of core benefits and clinical bottlenecks for oligonucleotide-, antibody-based therapeutics and traditional adjuvants.

Type	Core Advantages	Clinical Bottlenecks/Limitations
Oligonucleotide Immunotherapeutics (CpG, STING agonists, etc.)	Potent innate immune activation, amplifies adaptive immunity; leading novel vaccine-adjuvant candidate [1]	Prone to degradation, short half-life; systemic use triggers non-specific immune activation and severe off-target toxicity, limiting clinical use [4]
Monoclonal-Antibody (mAb) Immunotherapy	Precise diseased cell targeting; core therapy for tumours/infections; some activate immune effector cells [5]	Weak intrinsic innate immune activation; cannot drive sustained high-intensity innate-adaptive immune cascades alone [7]
Traditional Adjuvants (Aluminum adjuvants, broad-spectrum nucleic-acid adjuvants, etc.)	Can effectively activate immune responses.	No targeting ability; non-specific stimulation, with local inflammation and systemic side effect risks [8]

**Table 2:** **Preclinical comparison of representative vaccine adjuvants.** Immunological performance, safety profiles and manufacturing features of aluminum adjuvant, free CpG and targeted AOC adjuvant.

Adjuvant Type	Immune-Response Bias	Targeting Capability	Systemic Safety	Manufacturing Complexity	Key Characteristics and Limitations
Aluminum Adjuvant	Induces Th2 humoral immunity, poor cellular immunity activation [56]	No targeting ability	Local inflammation and systemic side effect risks	Simple process, low cost	Strengths: Effective immune activation; Limitations: Single immune profile, no targeting, high side effect risk
Free CpG Adjuvant	Induces Th1 immune responses, no targeting selectivity	No targeting, induces broad systemic activation	High systemic toxicity, off-target inflammation risk	Simple process, relatively low cost	Strengths: Potent innate immune activation; Limitations: Poor targeting, narrow safety window, high systemic administration risk
Targeted AOC Adjuvant	Activates Th1 cellular and humoral immunity simultaneously	Precise targeting, selective delivery to immune subsets	Preclinical data suggest local immune signal enrichment with lower systemic inflammation; superior safety is hypothesized but not yet demonstrated in comparative human studies	Complex site-specific conjugation process, high cost	Strengths: Strong targeting, potent immune activation in preclinical models, improved safety profile in preclinical studies [57,58]; Limitations: Complex process, high cost, long-term in vivo stability needs verification

**Table 3:** **Comparison of AOCs, free oligonucleotides and unconjugated antibodies.** The comparison reflects the published evidence discussed in this review. Direct head-to-head data are scarce.

Parameter	Free Oligonucleotide	Unconjugated Antibody	AOC
Cellular uptake	Poor and non-specific	Receptor-mediated; target-cell specific	Antibody-directed; target-cell specific
Immune-cell specificity	Low; broad activation	Depends on the target antigen	High; payload delivered to selected immune cells
Pharmacokinetics	Rapid nuclease degradation; short half-life	Long half-life	Intermediate; depends on linker stability
Immune activation	Strong innate activation via TLR and cytosolic sensors	Weak intrinsic innate activation	Targeted innate activation driven by the payload
Cytokine production	Systemic; dose-limiting	Minimal	Localized; lower systemic exposure in models
Toxicity	CRS; off-target inflammation	Infusion reactions; immunogenicity	Payload shedding; Fc-mediated off-target activation
Therapeutic window	Narrow	Wide for tolerated targets	Under evaluation; promising in preclinical studies

**Table 4:** **Predicted reprogramming of immune-cell metabolic pathways by AOCs.** Mechanisms and molecular targets are inferred from analogous TLR-agonist and ADC studies; direct AOC-specific metabolic evidence is currently lacking.

Metabolic Pathway	Core Regulatory Mechanism	Key Molecular Targets	Functional Effects
Glycolysis Pathway	Inferred pathway: internalized oligonucleotide payload may activate TLR-NF-kappaB signalling and upregulate HIF-1alpha, shifting cells toward aerobic glycolysis. Analogous effects shown with free TLR agonists; not yet confirmed with intact AOC constructs [83].	TLR-NF-κB, HIF-1α metabolic transcription factors	Boosts glucose uptake and lactate production, switches immune-cell metabolic pattern for activation.
Lipid Metabolism Pathway	Predicted effect: immune-stimulatory signals may remodel intracellular fatty acid synthesis/oxidation balance. Evidence derived from NLRP3/inflammasome studies, not AOC-specific experiments	Regulatory molecules related to fatty acid synthesis/oxidation	Restores immune-cell lipid homeostasis, adapts to material/energy needs post-activation.
Glutamine Metabolism Pathway	Predicted effect: immune-stimulatory signals may upregulate glutamine transporters and enhance glutamine catabolism. Analogous observations reported in macrophage and neutrophil metabolism; no AOC-specific metabolic flux data exist	Glutamine transporters	Increases glutamine uptake and catabolism, provides nitrogen, energy and anabolic precursors for immune-cell activation.

**Table 5:** **Regulatory status of representative AOC candidates (verified against FDA sources, October 2026).** None of the listed candidates has been approved by the FDA. All AOCs remain investigational. The immunomodulatory AOCs discussed in this review are all at the preclinical stage.

Candidate	Sponsor	Antibody Target	Oligonucleotide Payload	Indication	Development Stage
AOC 1001 (del-desiran)	Avidity Biosciences	Anti-TfR1	DMPK-targeting siRNA	Myotonic dystrophy type 1	Phase 3 (HARBOR)
AOC 1044	Avidity Biosciences	Anti-TfR1	Exon 44-skipping ASO	Duchenne muscular dystrophy	Phase 1/2 (EXPLORE44)
DYNE-101 (z-basivarsen)	Dyne Therapeutics	Anti-TfR1 Fab	DMPK-targeting ASO	Myotonic dystrophy type 1	Phase 1/2 (ACHIEVE); Phase 3 (HARMONIA) initiated

**Table 6:** **Evidence summary of the three immunomodulatory applications of AOCs.** Evidence levels are assigned according to the sources cited in the corresponding sections.

Application	AOC Composition (Examples)	Target Cell/Receptor	Oligonucleotide Payload	Immune Mechanism	Main Findings	Evidence Level
Vaccine adjuvant	CLEC9A-targeted AOC-CpG	Conventional type-1 dendritic cells/CLEC9A	CpG-ODN	TLR9 activation; DC maturation; Th1 responses	Localized immune stimulation with reduced systemic activation [107]	Preclinical (in vitro, mouse models)
Neutrophil-driven innate activation	Neutrophil-receptor-targeted AOCs	Neutrophils	TLR-stimulating oligonucleotides	TLR-dependent activation; recruitment; NET release; DC crosstalk	Enhanced early innate defence; tumour microenvironment remodelling [108]	Preclinical (in vitro, mouse models)
Immunometabolic reprogramming	AOC-TLR agonist conjugates	Dendritic cells, neutrophils, macrophages	Immunostimulatory oligonucleotides	TLR-NF-κB-HIF-1α axis; glycolysis, lipid and glutamine remodelling	Proposed sustained adjuvant effects [109]	Proposed mechanism; limited direct AOC data

## Data Availability

No new data were created or analyzed in this study. Data sharing is not applicable to this article.
